# Supplementary material for: Morphological Characterization of Root System Architecture in Diverse Tomato Genotypes during Early Growth
Source: Int J Mol Sci. 2018 Dec 5;19(12):3888. doi: 10.3390/ijms19123888 (PMC6321557; doi:10.3390/ijms19123888)
Supplement: Supplementary file 1 [file ijms-19-03888-s001.zip › 0 - Supplementary Files_legends_reviewed.pdf]

Supplementary Files

Figure S1. Early growth variation of selected tomato genotypes. (**a**) Experimental design for the evaluation of early RSA in tomato. Seeds were cold stored for 2 days and germinated for 3 days in a humid chamber (24 h darkness at 28ºC) before being transferred to half-Murashige and Skoog plates in a growth chamber (16 h light 26±1°C and 8 h darkness 23±1°C). The PR tips were excised 3 days after sowing (3 das) to induce LR formation, which were scored 6 das. Subsequently, the whole root was excised to induce AR formation from the hypocotyl that were scored at 6 and 12 days after AR induction (dai), corresponding to 12 and 18 das. In parallel, at 10 das, the seedlings were transferred to new plates to measure AR initiation and AR growth rates. (**b**) The germination percentages of wild tomato species, commercial tomato cultivars, and developmental mutants in the 'Micro-Tom' background between 1 and 4 days. (**c**) Lengths of PR at 0 and 3 days. Average ± SD values are shown. Different letters indicate significant differences (LSD; p-value<0.01) over genotypes.

Figure S2. Variation of some LR traits in the studied genotypes. (**a**) Scatter plots of LR number according to PR length in wild tomato species and commercial tomato cultivars. (**b**) Percentage of consecutive LRs arising on the same side of the PR. Clusters from 2 to ≥6 LRs with the same growth direction are indicated. (**c**) Median values of the LR growth vector. Different letters indicate significant differences (LSD; p-value<0.01) over genotypes.

Figure S3. RSA after root tip excision in (**a**) wild tomato species and (**b**) commercial tomato cultivars. Arrowheads point to the root-hypocotyl junction, and the asterisks indicate the distal end of the PR after excision. Scale bar: 10 mm.

Figure S4. LR distribution in the studied genotypes. (**a-c**) Average percentages of LR distribution along the length of the PR in (**a**) wild tomato species, (**b**) commercial tomato cultivars, and (**c**) developmental mutants in the 'Micro-Tom' genetic background. Different letters indicate significant differences (LSD; p-value<0.01) with regard to PR length (i.e., root depth).

Figure S5. LR length distribution in the studied genotypes. (**a**) Histograms for LR length in wild tomato species and commercial tomato cultivars (left), as well as in 'Micro-Tom' developmental mutants (right), with an overlay of the theoretical gamma distribution. (**b-d**) Histograms for LR length in (**b**) wild tomato species, (**c**) commercial tomato cultivars, and (**d**) 'Micro-Tom' developmental mutants.

Figure S6. RSA after PR tip excision in 'Micro-Tom' developmental mutants. Arrowheads point to the root-hypocotyl junction, and the asterisks indicate the distal end of the PR after excision. Scale bar: 10 mm.

Figure S7. ARs in the hypocotyl after whole root excision of selected tomato genotypes. (**a**) Wild tomato species, (**b**) some commercial tomato cultivars, and (**c**) 'Micro-Tom' developmental mutants. Scale bars: 10 mm.

Figure S8. Variation of some AR traits after whole root excision. (**a**) Average initiation time of consecutive ARs (first, second and third); dai: days after AR induction. (**b**) AR response estimated as the percentage of hypocotyl explants showing emergence of ARs at the indicated times. Asterisks indicate significant differences (LSD; p-value<0.01) over genotypes. (**c-d**) Median values of (**c**) AR length and (**d**) AR growth angle. Different letters indicate significant differences (LSD; p-value<0.01) over genotypes (**c**, **d**).

Figure S9. ARs in the hypocotyl after whole root excision of selected tomato genotypes. Scale bar: 10 mm.

Table S1. RSA traits measured in this study.

Table S2. Classification of the studied tomato genotypes based on early RSA attributes.

Table S3. Individual data values obtained in this study.
