# Supplementary material for: Morphological Characterization of Root System Architecture in Diverse Tomato Genotypes during Early Growth
Source: Int J Mol Sci. 2018 Dec 5;19(12):3888. doi: 10.3390/ijms19123888 (PMC6321557; doi:10.3390/ijms19123888)
Supplement: Supplementary file 1 [file ijms-19-03888-s001.zip › Figure_S1.pdf]

Figure S1

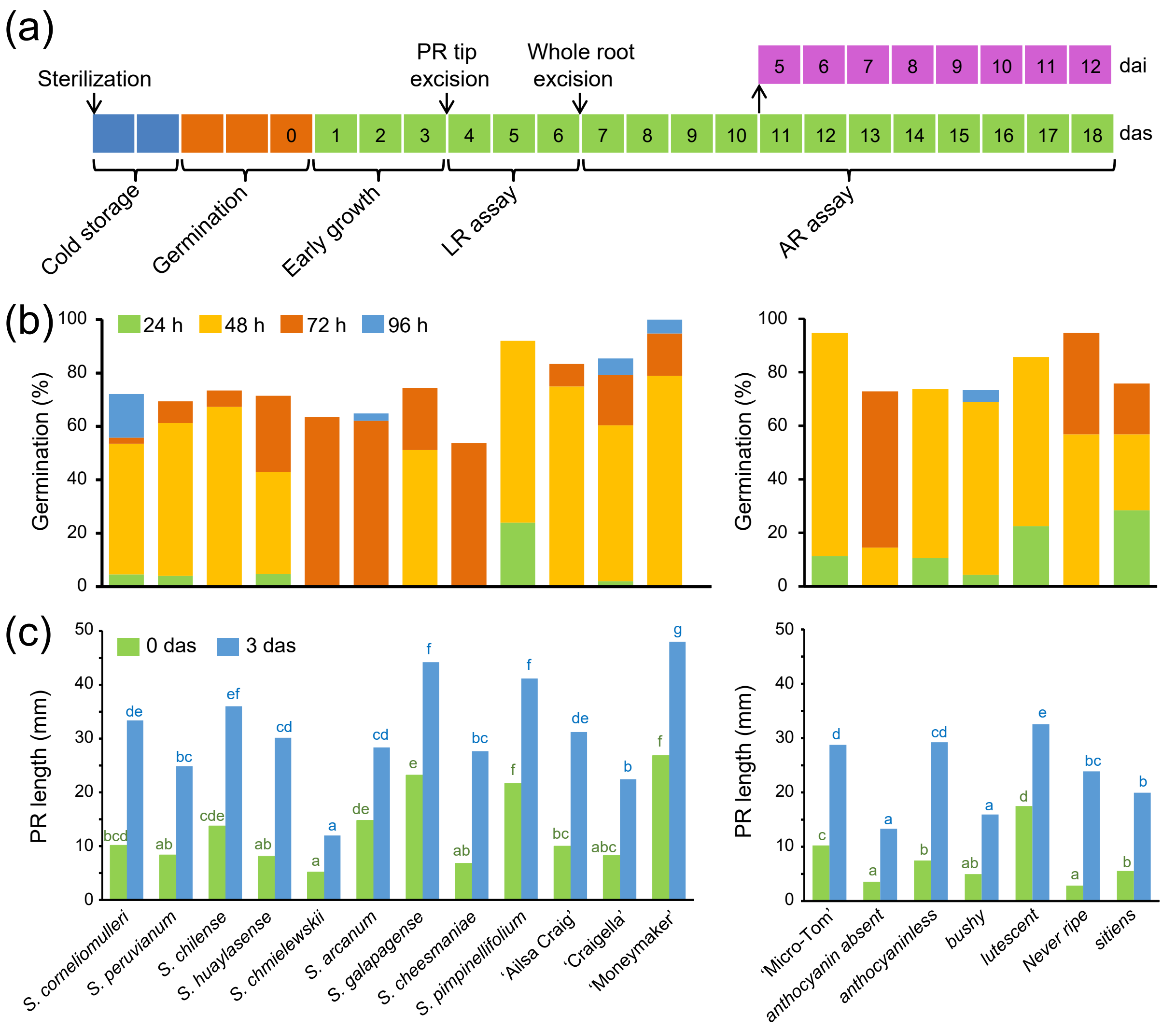

Figure S1. Early growth variation of selected tomato genotypes. (**a**) Experimental design for the evaluation of early RSA in tomato. Seeds were cold stored for 2 days and germinated for 3 days in a humid chamber (24 h darkness at 28ºC) before being transferred to half-Murashige and Skoog plates in a growth chamber (16 h light 26±1°C and 8 h darkness 23±1°C). The PR tips were excised 3 days after sowing (3 das) to induce LR formation, which were scored 6 das. Subsequently, the whole root was excised to induce AR formation from the hypocotyl that were scored at 6 and 12 days after AR induction (dai), corresponding to 12 and 18 das. In parallel, at 10 das, the seedlings were transferred to new plates to measure AR initiation and AR growth rates. (**b**) The germination percentages of wild tomato species, commercial tomato cultivars, and developmental mutants in the 'Micro-Tom' background between 1 and 4 days. (**c**) Lengths of PR at 0 and 3 days. Average ± SD values are shown. Different letters indicate significant differences (LSD; p-value<0.01) over genotypes.
