# Supplementary material for: Morphological Characterization of Root System Architecture in Diverse Tomato Genotypes during Early Growth
Source: Int J Mol Sci. 2018 Dec 5;19(12):3888. doi: 10.3390/ijms19123888 (PMC6321557; doi:10.3390/ijms19123888)
Supplement: Supplementary file 1 [file ijms-19-03888-s001.zip › Figure_S2.pdf]

Figure S2

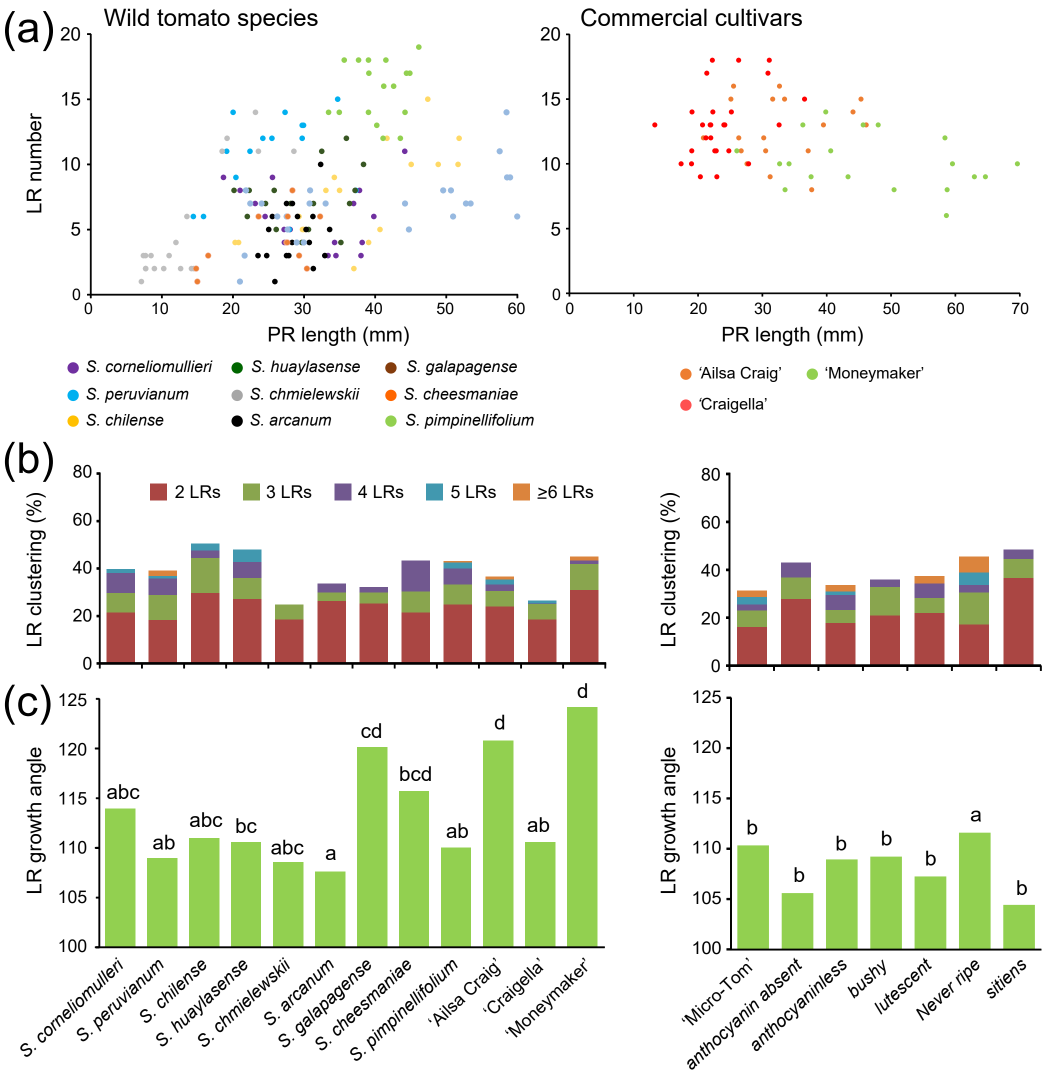

Figure S2. Variation of some LR traits in the studied genotypes. (**a**) Scatter plots of LR number according to PR length in wild tomato species and commercial tomato cultivars. (**b**) Percentage of consecutive LRs arising on the same side of the PR. Clusters from 2 to ≥6 LRs with the same growth direction are indicated. (**c**) Median values of the LR growth vector. Different letters indicate significant differences (LSD; p-value<0.01) over genotypes.
