# Supplementary material for: Morphological Characterization of Root System Architecture in Diverse Tomato Genotypes during Early Growth
Source: Int J Mol Sci. 2018 Dec 5;19(12):3888. doi: 10.3390/ijms19123888 (PMC6321557; doi:10.3390/ijms19123888)
Supplement: Supplementary file 1 [file ijms-19-03888-s001.zip › Figure_S4.pdf]

Figure S4

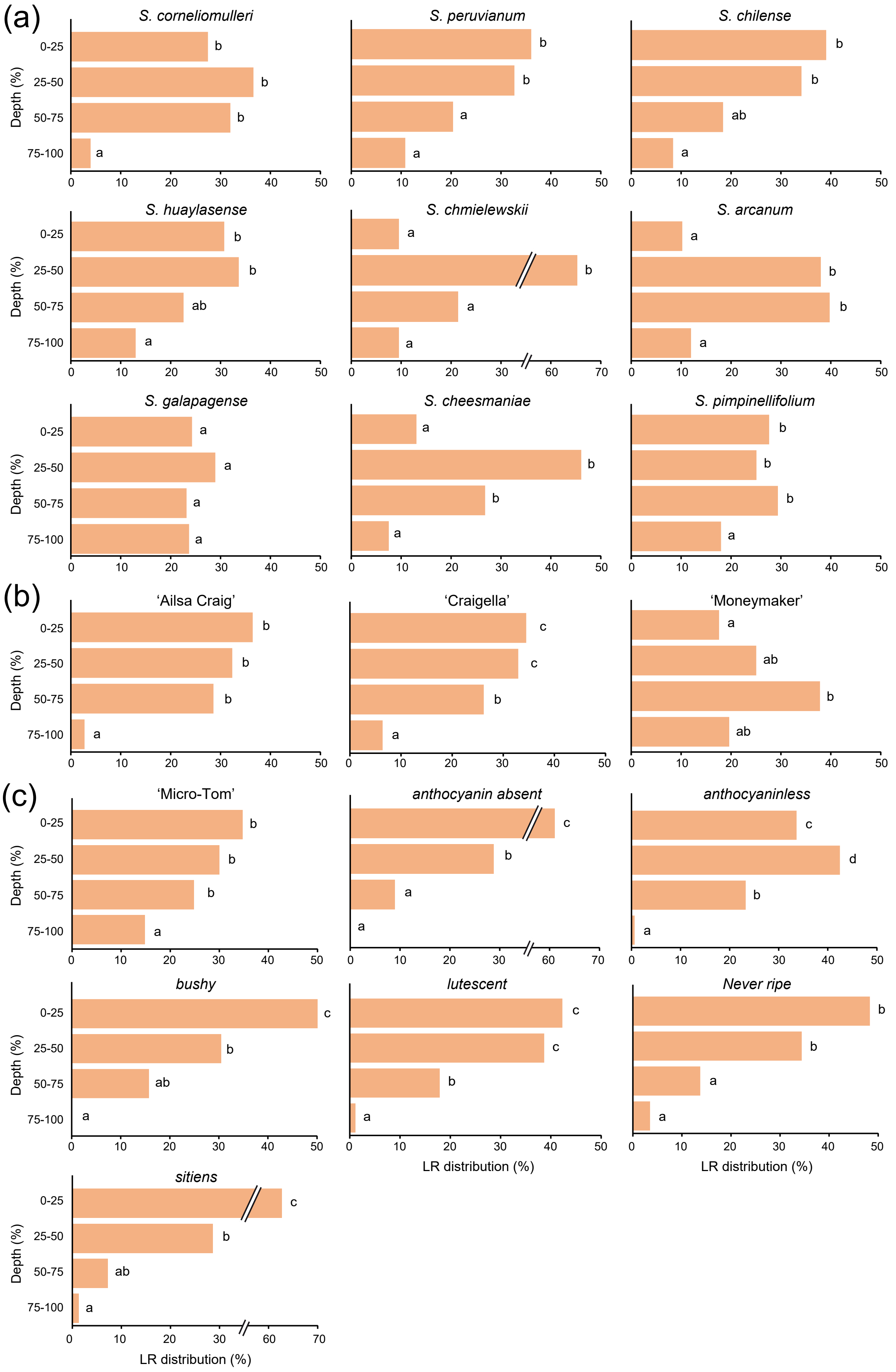

Figure S4. LR distribution in the studied genotypes. (**a-c**) Average percentages of LR distribution along the length of the PR in (**a**) wild tomato species, (**b**) commercial tomato cultivars, and (**c**) developmental mutants in the 'Micro-Tom' genetic background. Different letters indicate significant differences (LSD; p-value<0.01) with regard to PR length (i.e., root depth).
