# Supplementary material for: Morphological Characterization of Root System Architecture in Diverse Tomato Genotypes during Early Growth
Source: Int J Mol Sci. 2018 Dec 5;19(12):3888. doi: 10.3390/ijms19123888 (PMC6321557; doi:10.3390/ijms19123888)
Supplement: Supplementary file 1 [file ijms-19-03888-s001.zip › Figure_S5.pdf]

Figure S5

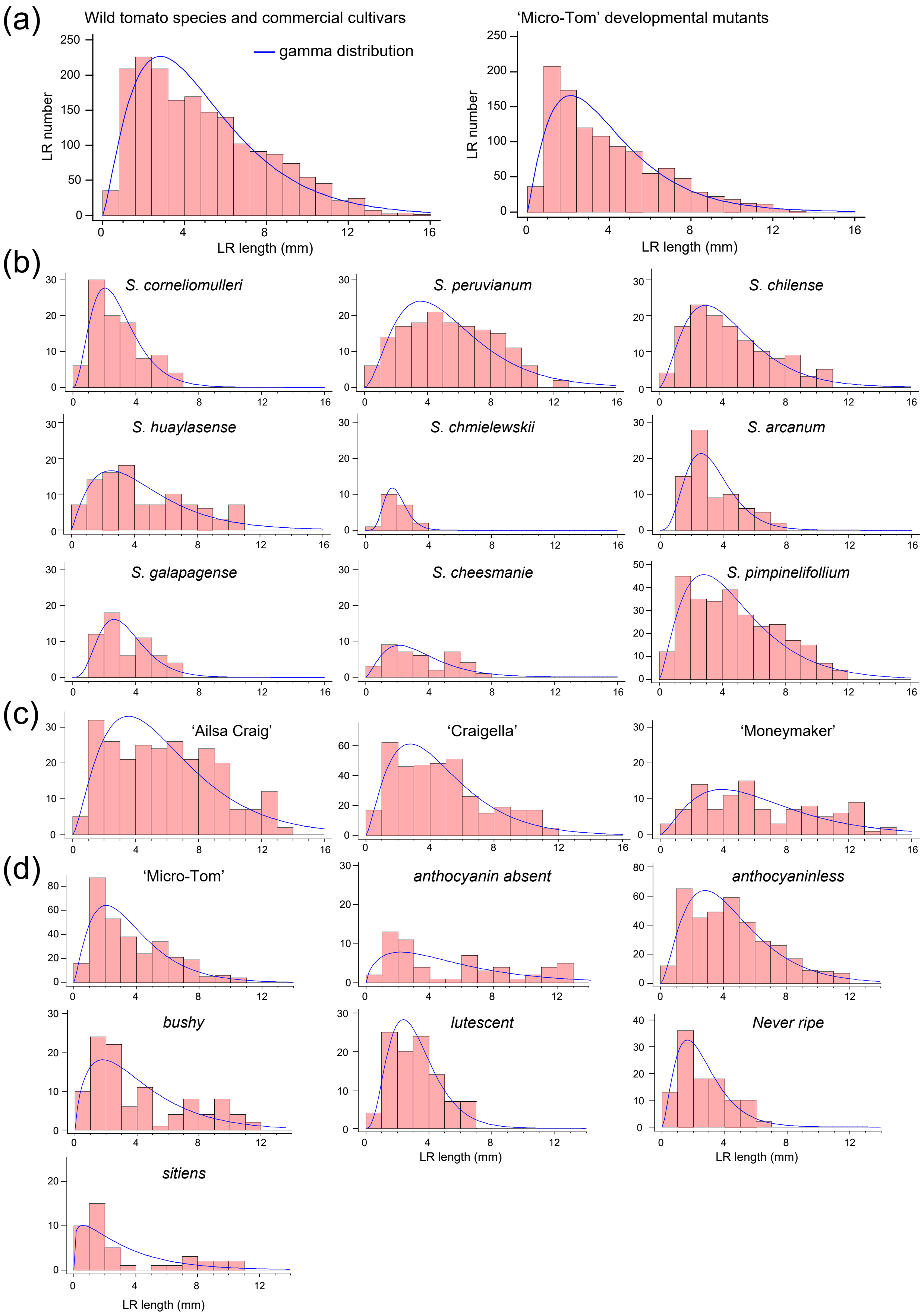

Figure S5. LR length distribution in the studied genotypes. (**a**) Histograms for LR length in wild tomato species and commercial tomato cultivars (left), as well as in 'Micro-Tom' developmental mutants (right), with an overlay of the theoretical gamma distribution. (**b-d**) Histograms for LR length in (**b**) wild tomato species, (**c**) commercial tomato cultivars, and (**d**) 'Micro-Tom' developmental mutants.
