# Supplementary material for: Morphological Characterization of Root System Architecture in Diverse Tomato Genotypes during Early Growth
Source: Int J Mol Sci. 2018 Dec 5;19(12):3888. doi: 10.3390/ijms19123888 (PMC6321557; doi:10.3390/ijms19123888)
Supplement: Supplementary file 1 [file ijms-19-03888-s001.zip › Figure_S6.pdf]

Figure S6

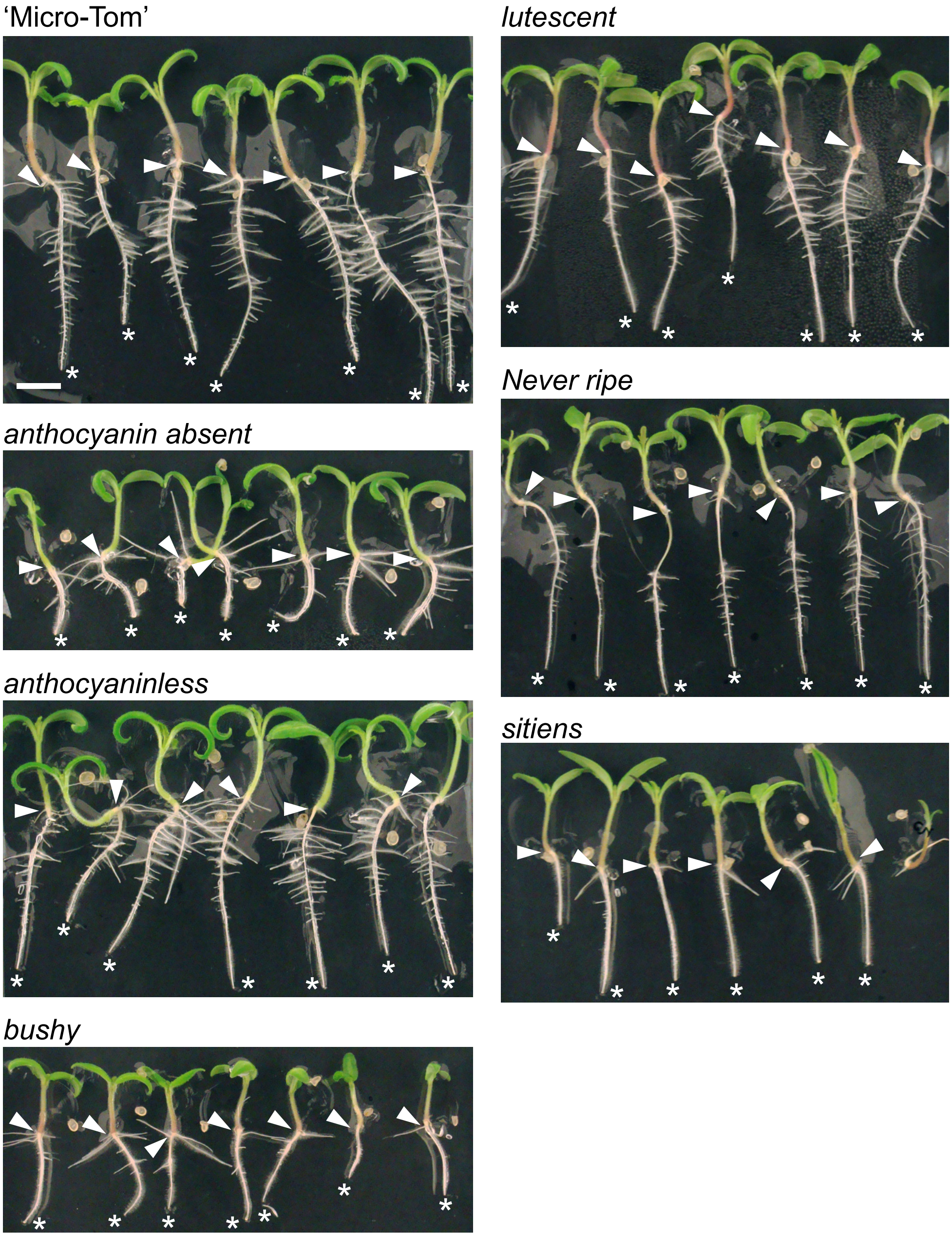

Figure S6. RSA after PR tip excision in 'Micro-Tom' developmental mutants. Arrowheads point to the root-hypocotyl junction, and the asterisks indicate the distal end of the PR after excision. Scale bar: 10 mm.
