# Supplementary material for: Morphological Characterization of Root System Architecture in Diverse Tomato Genotypes during Early Growth
Source: Int J Mol Sci. 2018 Dec 5;19(12):3888. doi: 10.3390/ijms19123888 (PMC6321557; doi:10.3390/ijms19123888)
Supplement: Supplementary file 1 [file ijms-19-03888-s001.zip › Figure_S7.pdf]

Figure S7

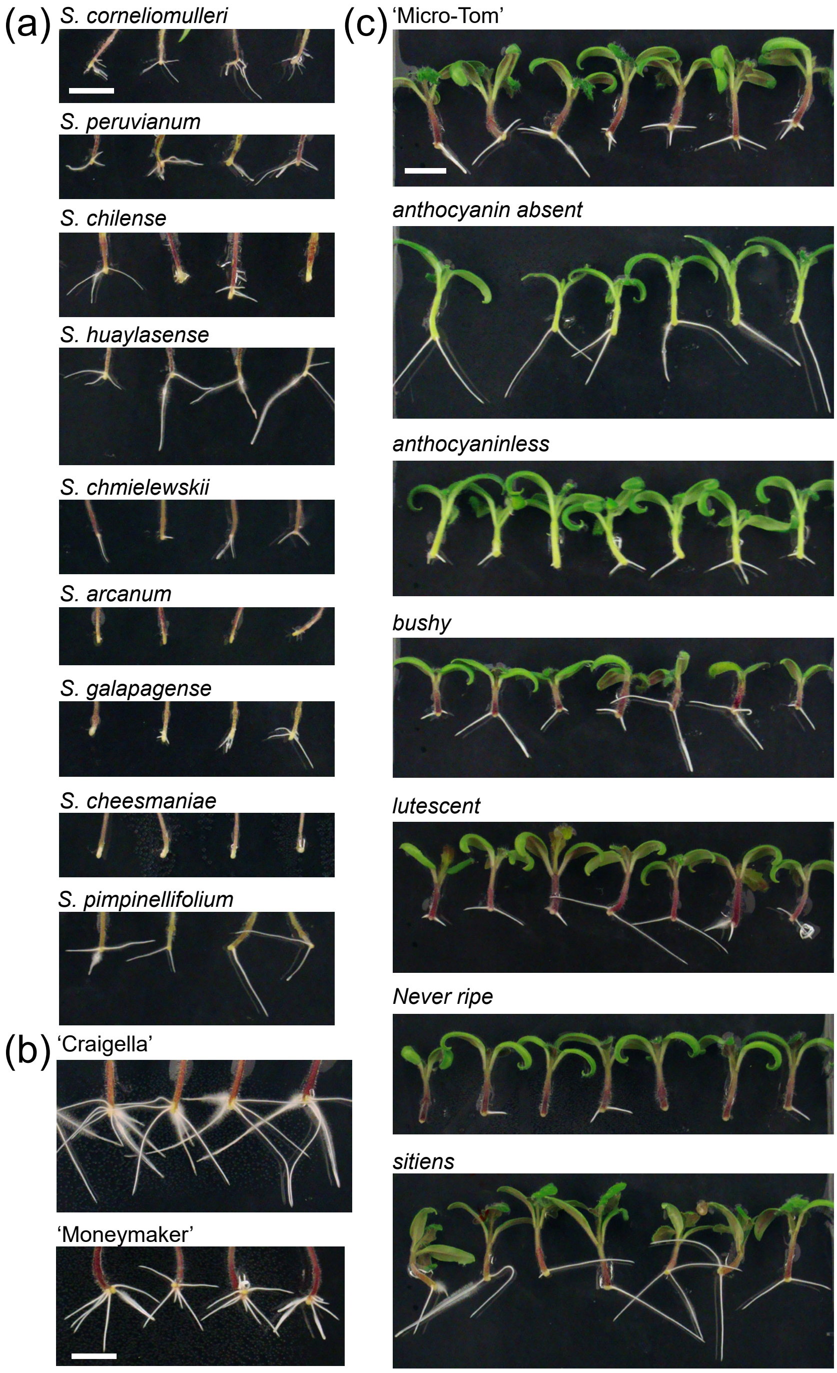

Figure S7. ARs in the hypocotyl after whole root excision of selected tomato genotypes. (**a**) Wild tomato species, (**b**) some commercial tomato cultivars, and (**c**) 'Micro-Tom' developmental mutants. Scale bars: 10 mm.
