# Supplementary material for: Morphological Characterization of Root System Architecture in Diverse Tomato Genotypes during Early Growth
Source: Int J Mol Sci. 2018 Dec 5;19(12):3888. doi: 10.3390/ijms19123888 (PMC6321557; doi:10.3390/ijms19123888)
Supplement: Supplementary file 1 [file ijms-19-03888-s001.zip › Figure_S8.pdf]

Figure S8

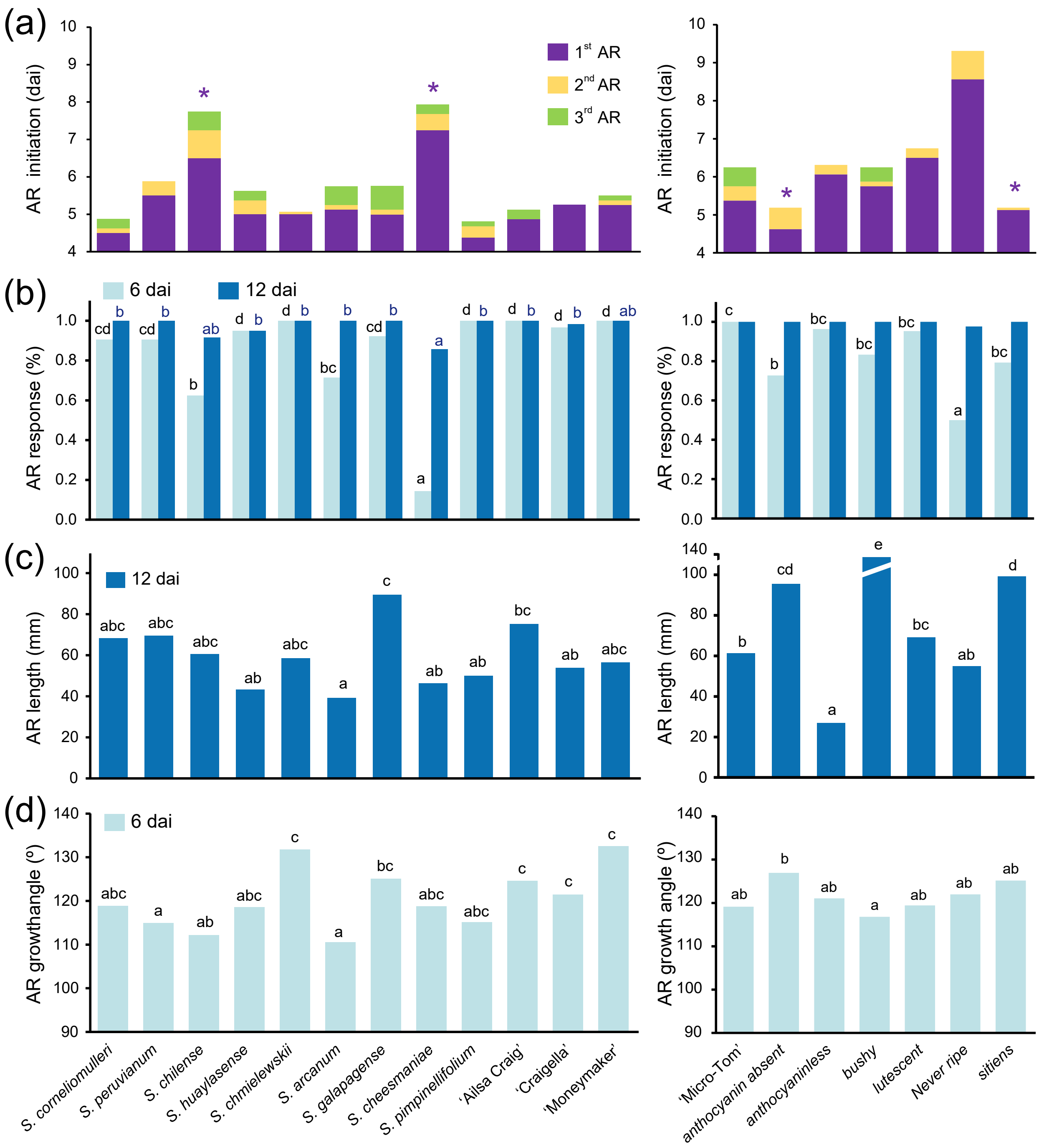

Figure S8. Variation of some AR traits after whole root excision. (**a**) Average initiation time of consecutive ARs (first, second and third); dai: days after AR induction. (**b**) AR response estimated as the percentage of hypocotyl explants showing emergence of ARs at the indicated times. Asterisks indicate significant differences (LSD; p-value<0.01) over genotypes. (**c-d**) Median values of (**c**) AR length and (**d**) AR growth angle. Different letters indicate significant differences (LSD; p-value<0.01) over genotypes (**c**, **d**).
