# Supplementary material for: Morphological Characterization of Root System Architecture in Diverse Tomato Genotypes during Early Growth
Source: Int J Mol Sci. 2018 Dec 5;19(12):3888. doi: 10.3390/ijms19123888 (PMC6321557; doi:10.3390/ijms19123888)
Supplement: Supplementary file 1 [file ijms-19-03888-s001.zip › Table_S1_NEW.pdf]

Table 1. RSA traits measured in this study.

Studied trait	Description	Measured
Germination (%)	Percentage of seeds with emerged radicle (>2 mm)	Between 1 and 4 days after cold storage
Primary root (PR) length (mm)	Average length of PR after sowing	0 days after sowing (das), 3 das
PR growth rate (mm/h)	Average increase of PR length between 3 and 0 das	-
Lateral root (LR) number	Average number of lateral roots	3 days after root tip excision (6 das)
LR distribution (%)	Average proportion of LRs along quartiles of PR length (i.e. depth)	6 das
LR distance (mm)	Median distance of two consecutive LRs	6 das
LR length (mm)	Median length of LRs	6 das
LR clustering (%)	Average percentage of consecutive LRs with the same growth direction	6 das
LR growth angle (º)	Average angle of LR growth vector as regards its PR growth vector	6 das
Adventitious root (AR) number	Average number of ARs	6 and 12 days after whole root excision (12 and 18 das)
AR growth rate (mm/h)	Average increase of AR length during the first 12 h after AR emergence	-
AR initiation time (h)	Average time from whole root excision until AR emergence	-
AR response (%)	Percentage of hypocotyls with emerged ARs	12 and 18 das
AR length (mm)	Median length of ARs	12 das
AR growth angle (º)	Average angle of AR growth vector as regards its hypocotyl growth vector	12 das
Maximum AR length (mm)	Maximum length of ARs	18 das
