# Supplementary material for: Morphological Characterization of Root System Architecture in Diverse Tomato Genotypes during Early Growth
Source: Int J Mol Sci. 2018 Dec 5;19(12):3888. doi: 10.3390/ijms19123888 (PMC6321557; doi:10.3390/ijms19123888)
Supplement: Supplementary file 1 [file ijms-19-03888-s001.zip › Table_S2.pdf]

Table S2. Classification of studied tomato genotypes based on early RSA attributes.

Genotype	PR growth	LR					AR				RSA ideotype
		Number	Distance	Length	Distribution	Growth angle	Number	Length	Growth angle	Growth rate	
S. corneliomulleri	C	A	B	B	B	B	A	C	B	B	1
S. peruvianum	B	B	B	B	B	B	B	B	B	B	4
S. chilense	C	B	C	B	B	B	A	A	B	B	1
S. huaylasense	C	B	B	B	B	B	B	A	B	B	1
S. chmielewskii	A	A	A	A	C	B	B	A	B	B	2
S. arcanum	B	A	C	B	C	B	C	B	B	B	3
S. galapagense	B	A	C	B	A	B	B	B	B	B	3
S. cheesmanie	B	A	B	B	C	B	B	A	B	B	3
S. pimpinelifollium	B	C	B	B	B	B	C	B	B	C	4
'Ailsa Craig'	C	B	B	B	B	B	A	B	B	B	4
'Craigella'	B	B	B	B	B	B	B	A	B	A	4
'Moneymaker'	B	B	B	B	C	C	B	B	B	B	5
'Micro-Tom'	B	C	B	B	B	B	C	B	B	C	6
anthocyanin absent	A	A	B	B	B	B	B	A	B	A	8
anthocyaninless	C	C	B	B	B	B	A	B	B	B	6
bushy	A	B	B	B	B	B	B	B	B	A	8
lutescent	B	B	B	B	B	B	C	B	B	B	6
Never ripe	B	B	B	A	B	B	A	A	B	B	7
sitiens	A	A	B	B	B	B	B	A	B	A	8
